# HFpEF Diagnosis: A Challenge in CKD with Current Algorithms

**DOI:** 10.3390/life16060944

**Published:** 2026-06-03

**Authors:** Anca E. Stefan, Maria A. Covic, Gianina Dodi, Alexandra E. Avanu, Silviu Bîrgoan, Corneliu Moroșanu, Amin Bazyani, Mehmet Kanbay, Radu A. Sascau, Adrian C. Covic

**Affiliations:** 1 Grigore T. Popa University of Medicine and Pharmacy Iasi, 700115 Iasi, Romania; anca-elena.stefan@umfiasi.ro (A.E.S.); gianina.dodi@umfiasi.ro (G.D.); gavril-silviu.birgoan@umfiasi.ro (S.B.); radu.sascau@umfiasi.ro (R.A.S.); adrian.covic@umfiasi.ro (A.C.C.); 2 Dr. C. I. Parhon Clinical Hospital, 700503 Iasi, Romania; corneliumorosanu@gmail.com; 3 Cardiovascular Diseases Institute Prof. Dr. George I.M. Georgescu, 700503 Iasi, Romania; 4 Division of Nephrology, Koc University School of Medicine, 34450 Istanbul, Turkey; aminbazyani@gmail.com (A.B.); mkanbay@ku.edu.tr (M.K.); 5 Academy of Romanian Scientists, 030171 Bucharest, Romania

**Keywords:** cardio-kidney-metabolic axis, chronic kidney disease heart failure, CKD HFpEF, CKD HFpEF metabolic risk, HFpEF CKD

## Abstract

Background: Chronic kidney disease (CKD) is associated with a high burden of cardiovascular remodeling and increased risk of heart failure with preserved ejection fraction (HFpEF). However, the interpretation of natriuretic peptide-based HFpEF diagnostic remains challenging in CKD populations, where structural cardiac abnormalities and elevated NT-proBNP levels frequently coexist. Methods: We conducted a cross-sectional study including ambulatory patients with CKD stages G3–G4 and NYHA II dyspnea. Clinical, metabolic, vascular, and echocardiographic assessments were performed. HFpEF was assessed using a modified HFA-PEFF-based approach derived from the ESC-recommended diagnostic algorithm. We evaluated the impact of NT-proBNP thresholds on HFpEF classification and explored the relationship between NT-proBNP, echocardiographic diastolic dysfunction, and structural cardiac abnormalities. Results: The cohort displayed a high cardiometabolic burden (74.9%), and structural cardiac abnormalities were highly prevalent. Using a modified HFA-PEFF diagnostic algorithm, HFpEF was identified in 52.9% of patients. However, when the biomarker domain was excluded, 86.7% of patients remained within the intermediate-probability range. In an exploratory analysis, a cutoff of 700 pg/mL was identified as the cohort-adapted threshold with the best diagnostic balance and identified 19.8% patients as having HFpEF. Conclusions: Patients with CKD G3–G4 exhibited substantial structural and functional cardiovascular abnormalities despite no prior diagnosis of heart failure. HFpEF classification varied according to the NT-proBNP threshold applied, while NT-proBNP demonstrated limited discriminatory performance for echocardiographic diastolic dysfunction. These findings support the need for more refined and CKD-sensitive approaches for HFpEF characterization in this population.

## 1. Introduction

Chronic kidney disease (CKD) is a major global health concern, affecting over 700 million people worldwide [1]. The prevalence of CKD increased exponentially since 1990, due to population growth, aging, and rising rates of diabetes mellitus and arterial hypertension [1,2]. CKD is a powerful and independent risk factor for cardiovascular (CV) and all-cause mortality. CV mortality is significantly increased in CKD, even in moderately reduced renal function. CV mortality risk is increased even in moderate CKD. In stage 3 CKD, the relative risk is approximately 1.5–2.8 with moderate albuminuria and rises to ~4.3–5.2 with severe albuminuria. This risk increases further in stage 4 CKD, reaching ~4.8–8.1, emphasizing the strong association between CKD severity and CV outcomes [3].

In terms of morbidity, CKD is associated with a broad spectrum of CV complications, including coronary artery disease, heart failure, arrhythmias, and sudden cardiac death. The relationship between CKD and CV disease is bidirectional: reduced kidney function promotes vascular and myocardial remodeling through chronic inflammation, oxidative stress, and bone mineral disorder, while CV pathologies such as heart failure (HF) can further accelerate renal function decline. This interplay leads to a progressive vicious cycle in which CKD acts both as a driver and a consequence of CV disease, thus amplifying cardiac and renal organ dysfunction, morbidity, and mortality [4].

HF represents a major cardiovascular complication in CKD. However, it remains frequently underrecognized in this population, particularly in its preserved ejection fraction phenotype. Prior studies have reported incidence rates of 0.9 (95% CI, 0.8–1.0) for heart failure with preserved ejection fraction (HFpEF) and 0.7 (95% CI, 0.6–0.8) per 100 person-years for heart failure with reduced ejection fraction (HFpEF) among patients with CKD [5].

At the same time, interpretation of natriuretic peptides in CKD remains challenging, as impaired renal function, myocardial remodeling, arterial stiffness, and volume-related factors may contribute to elevated NT-proBNP levels even in the absence of overt heart failure. Although NT-proBNP is a well-established prognostic biomarker in CKD and is strongly associated with cardiovascular outcomes, its role within current HFpEF diagnostic algorithms in CKD populations remains insufficiently characterized. Consequently, standard biomarker thresholds derived from the general population may substantially influence HFpEF classification in patients with impaired renal function [6]. Moreover, the newly proposed unifying concept of the cardiovascular-kidney-metabolic (CKM) syndrome shows the interconnected nature of diabetes or obesity, CKD, and CV disease as a pathophysiological continuum [7].

In this context, we conducted a cross-sectional study including patients with CKD stages G3–G4 and unexplained NYHA class II dyspnea, in whom we performed comprehensive metabolic, vascular, and echocardiographic phenotyping beyond conventional systolic function assessment. We aimed to characterize the burden of structural and diastolic cardiac abnormalities in this population and to evaluate the influence of NT-proBNP thresholds on HFpEF classification using the HFA-PEFF diagnostic algorithm.

## 2. Materials and Methods

### 2.1. Study Design and Population

This was a cross-sectional study that included adult patients with CKD stages 3 and 4, defined by an estimated glomerular filtration rate (eGFR) between 15 and 60 mL/min/1.73 m^2^, calculated using the CKD-EPI 2021 equation [8]. We selected patients during routine ambulatory visits at Dr. C.I. Parhon Clinical Hospital, Iasi, Romania. This study was registered on the ClinicalTrials.gov database with NCT07237451 identifier and approved by the Ethical Committee of Grigore T. Popa University of Medicine and Pharmacy of Iasi (no. 648/2025) and Dr. C.I. Parhon Hospital in Iasi, Romania (no. 1797/2024). All participants provided written informed consent prior to enrollment. Patients’ personal identification and information were encoded before being used for research. All experiments were performed in accordance with relevant guidelines and regulations.

### 2.2. Inclusion and Exclusion Criteria

Inclusion criteria were age > 18 years and a confirmed diagnosis of CKD stage 3 or 4, preserved left ventricular systolic function on echocardiography (defined as left ventricular ejection fraction—LEVF over 50%), grade II dyspnea according to the New York Heart Association (NYHA) classification [9], and euvolemic on clinical examination. Alternative major causes of dyspnea were clinically assessed and excluded based on medical history, physical examination, laboratory findings, and echocardiographic evaluation. Exclusion criteria integrated eGFR < 15 mL/min/1.73 m^2^ or ongoing dialysis, eGFR > 60 mL/min/1.73 m^2^, prior heart failure diagnosis, congenital heart disease, severe valvular disease on echocardiography, decompensated liver cirrhosis, pregnancy, active malignancy, and established coronary artery disease (defined as a history of acute or chronic coronary syndrome, previous invasive coronary angiography or CT angiography showing significant coronary lesions). Patients with implantable devices that would significantly impair cardiac imaging (including pacemakers or metallic joint prostheses) were also excluded.

### 2.3. Clinical and Laboratory Assessment

After we obtained informed consent, we performed a detailed medical history and review of current medication. We performed anthropometric measurements, including body weight, height, and abdominal circumference. Venous blood samples were collected for the assessment of serum creatinine, urea, electrolyte panel, hemoglobin levels, NT-proBNP, total cholesterol, LDL cholesterol, HDL cholesterol, triglycerides, and fasting plasma glucose. Urine samples were collected to assess proteinuria using the protein-to-creatinine ratio.

The serum concentration of N-terminal pro B-type natriuretic peptide (NT-proBNP) was determined using a commercially available sandwich enzyme-linked immunosorbent assay (ELISA) kit (NT-proBNP ELISA kit, cat. no. CSB-E05152h; Wuhan Huamei Biotech Co., Ltd., Wuhan, China). Blood samples were collected in gel and clot activator tubes at baseline. After coagulation, serum was separated by centrifugation (5 min at 4500 rpm), aliquoted into 0.5 mL tubes, and stored at −80 °C until analysis. All assays were performed according to the manufacturer’s instructions. Briefly, 100 µL of undiluted serum samples and corresponding standards were added to the microplate wells and incubated for 2 h at 37 °C using a Matrix Orbital Delta Plus incubator (IKA, Staufen, Germany). After removal of the liquid, 100 µL of biotin-conjugated antibody was added and incubated for 1 h at 37 °C. Wells were then washed, and 100 µL of horseradish peroxidase (HRP)–avidin conjugate was added, followed by another hour of incubation at 37 °C. After thorough washing, 90 µL of TMB (3,3′,5,5′-tetramethylbenzidine) substrate was added and incubated for 25 min in the dark. The reaction was stopped with 50 µL of stop solution. Optical density (OD) was measured at 450 nm, with wavelength correction at 540 and, subsequently, 570 nm, using a microplate reader (BMG Labtech SPECTRO star Nano microplate reader, Ortenberg, Germany, and SPECTRO star Nano V6.20 software). All samples and standards were analyzed in triplicate, and mean values were used for analysis. NT-proBNP concentrations were calculated from the standard curve, generated using four-parameter logistic (4-PL) regression (MyAssays online version R10.2 software), with a detection range of 0.313–20 ng/mL. The intra-assay and inter-assay coefficients of variation were <10% and <8%, respectively.

### 2.4. Metabolic Syndrome Assessment

Body mass index (BMI) was calculated as body weight (in kilograms) divided by the square of height (in meters) (kg/m^2^). Metabolic syndrome was defined according to the National Cholesterol Education Program Adult Treatment Panel III (NCEP ATP III) criteria [10].

### 2.5. Echocardiographic Assessment

Transthoracic echocardiography was performed using Philips CX50 or EPIQ 5 systems in patients with adequate acoustic windows. Cardiac structure and function were assessed according to American Society of Echocardiography recommendations [11]. The echocardiography protocol is detailed in Tables S1 and S2. A key limitation is the reliance on transthoracic echocardiography, which depends on acoustic window quality and is subject to observer variability, leading to missing data in some patients. Derived parameters were calculated according to current American Society of Echocardiography and European Society of Cardiology recommendations (Table 1) [11,12].

### 2.6. Pulse Wave Velocity Assessment

Arterial stiffness was measured through applanation tonometry of the carotid and femoral arteries using SphygmoCorTM; PWV Inc., Westmead, Sydney, Australia. Measurements were performed after patients lay at rest in the supine position for at least 10 min. Carotid and femoral artery pulse waves were measured. After measuring the transit time from the R-wave of the electrocardiogram to the foot of the carotid-femoral pulse, and the transit time measured between the feet of the two waveforms, we calculated carotid-femoral PWV as the difference between these two transit times divided by the duration of time needed for the pulse wave to travel the arterial path length.

### 2.7. Diagnosis of HFpEF

HFpEF was assessed using a guideline-based approach derived from ESC recommendations, incorporating functional, morphological, and biomarker domains (HFA-PEFF algorithm). Diagnosis was based on the presence of symptoms, preserved left ventricular ejection fraction, and objective evidence of diastolic dysfunction and/or elevated filling pressures. Echocardiographic parameters were evaluated according to current ASE/EACVI recommendations. The criteria used for HFpEF assessment are summarized in Table 1 [13]. Global longitudinal strain (GLS), a minor criterion for diagnosis, was excluded from the HFpEF diagnostic algorithm to ensure broader applicability, as strain imaging is not universally available. HFpEF classification in our study was based on a modified HFA-PEFF-oriented diagnostic framework and was not confirmed by invasive hemodynamic assessment or exercise testing.

To account for the specific characteristics of CKD, NT-proBNP was analyzed using both guideline-recommended thresholds and cohort-adapted cutoffs. Cohort-adapted cutoffs were explored based on their sensitivity and specificity for identifying echocardiographic diastolic dysfunction, and the threshold providing the best balance between sensitivity and specificity was selected.

### 2.8. End Points

The primary objective of this study was to assess the prevalence of HFpEF using the modified HFA-PEFF algorithm in patients with CKD G3–G4 using a guideline-based diagnostic approach and to evaluate the impact of NT-proBNP thresholds on HFpEF classification in this population.

Secondary objectives included assessing the relationship between NT-proBNP and echocardiographic diastolic dysfunction, evaluating the discriminatory performance of NT-proBNP for identifying diastolic dysfunction, and evaluating alternative cohort-derived NT-proBNP thresholds within the HFA-PEFF framework as an exploratory outcome.

### 2.9. Statistical Analysis

Statistical analyses were performed using JASP version 0.19.6 (JASP Team, University of Amsterdam). Continuous variables were tested for normality using the Shapiro–Wilk test and are presented as mean ± standard deviation or median (interquartile range), as appropriate. Categorical variables are reported as counts and percentages. Group comparisons were performed using the Mann–Whitney U test, Kruskal–Wallis test, or chi-squared test, as appropriate. HFpEF classification was performed using the HFA-PEFF diagnostic algorithm according to ESC-recommended thresholds. Exploratory analyses using cohort-adapted NT-proBNP thresholds were additionally performed. Associations between NT-proBNP and echocardiographic diastolic dysfunction were evaluated using binary logistic regression with log-transformed NT-proBNP values. Diagnostic performance was assessed using ROC analysis with calculation of the area under the curve (AUC), sensitivity, specificity, and accuracy. Sensitivity analyses were performed using alternative echocardiographic definitions of diastolic dysfunction. ROC analyses evaluated the discriminatory performance of NT-proBNP for identifying echocardiographic diastolic dysfunction rather than definitively adjudicated HFpEF. Given the absence of invasive hemodynamic confirmation and the complexity of establishing a certain HFpEF diagnosis in CKD populations, echocardiographic diastolic dysfunction was selected as a more objective and consistently assessable surrogate marker of HFpEF-related cardiac dysfunction. A two-sided *p*-value < 0.05 was considered statistically significant.

## 3. Results

### 3.1. Patient Characteristics

A total of 200 patients were screened, and 13 were excluded due to baseline LVEF < 50%. The final cohort included 187 patients with CKD stages G3–G4, with a median age of 68 years (IQR 59–75); 49.7% were male. The cohort displayed a marked cardiometabolic burden, with metabolic syndrome present in 74.9% of patients and increased arterial stiffness reflected by a median pulse wave velocity of 10.45 m/s (IQR 8.8–12.7). Hypertensive and diabetic nephropathy represented the leading etiologies of CKD. Median NT-proBNP was 405.5 pg/mL (IQR 122.1–841.4), without significant correlation with eGFR (r = −0.102, *p* = 0.166) or differences across CKD stages (*p* = 0.407). Additional baseline characteristics are summarized in Table 2.

### 3.2. Echocardiographic Measurements

**Figure 1:**
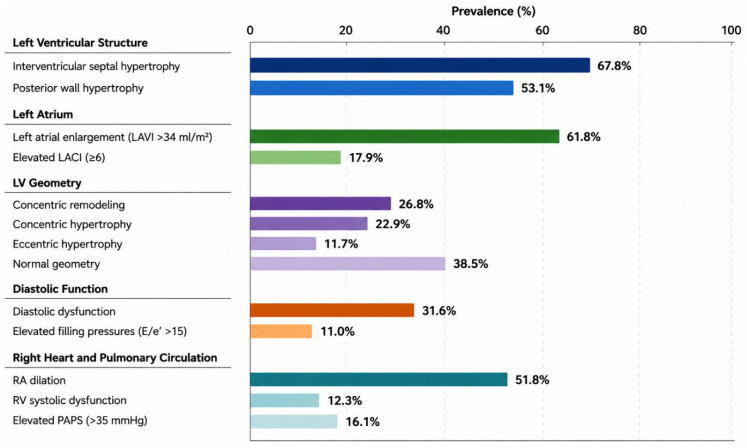
Prevalence of Structural and Functional Cardiac Abnormalities.

Structural cardiac abnormalities were highly prevalent in the cohort, particularly interventricular septal hypertrophy (67.8%), left atrial enlargement (61.8%), and right atrial dilation (51.8%). Concentric remodeling and hypertrophy were also frequent, while diastolic dysfunction was present in 31.6% of patients. Detailed echocardiographic findings are presented in Table 3 and Figure 1.

LV hypertrophy was defined using sex-specific LVMI cut-offs (≥95 g/m^2^ in women and ≥115 g/m^2^ in men). LVMI (left ventricular mass index) was calculated as LVM/BSA using the Devereux-modified ASE formula. RWT (relative wall thickness) was calculated as 2 × LVPWd/LVEDD. LV geometry was classified as concentric hypertrophy (increased LVMI and RWT > 0.42), concentric remodeling (normal LVMI and RWT > 0.42), eccentric hypertrophy (increased LVMI and RWT ≤ 0.42), and normal geometry (normal LVMI and RWT ≤ 0.42). LV dilatation was defined according to sex-specific LVEDV (left ventricular end-diastolic volume) cut-offs. Elevated filling pressures were defined as E/e′ > 15. Left atrial enlargement was defined as LAVI (left atrial volume index) > 34 mL/m^2^; LACI (left atrial coupling index) was calculated as LAVI divided by medial a’. RV (right ventricular) systolic dysfunction was defined as TAPSE (tricuspid annular plane systolic excursion) < 17 mm, FAC (fractional area change) < 35%, or S’ < 9.5 cm/s. PAPS (pulmonary artery systolic pressure) was calculated as 4 × (TR Vmax)^2^ + RAP (right atrial pressure) and considered elevated at >35 mmHg. Percentages were calculated based on available data. LV geometry (*n* = 2).

### 3.3. Primary Outcome

**Figure 2:**
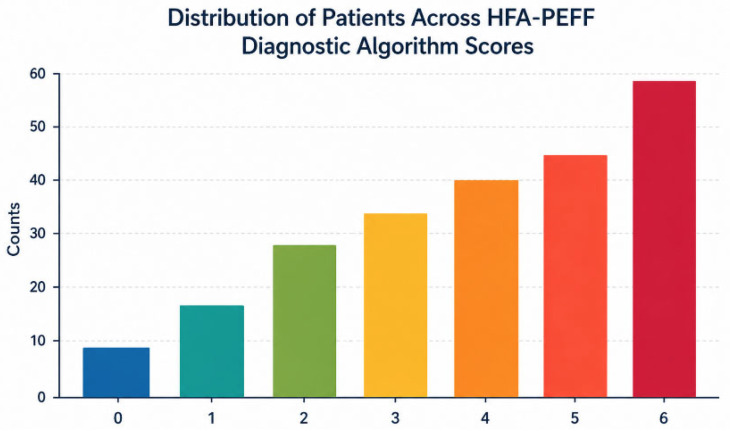
Distribution across modified HFA-PEFF Diagnostic Algorithm Scores.

After applying the modified ESC diagnostic criteria at the guideline thresholds (2 points for NT-proBNP > 220 pg/mL in sinus rhythm and >660 pg/mL in atrial fibrillation and 1 point for NT-proBNP 125–220 in sinus rhythm and 365–660 in atrial fibrillation), HFpEF was identified in 52.9% of patients (*n* = 99), while 47.1% (*n* = 88) did not meet the diagnostic criteria (Figure 1, Table 3). Among HFA-PEFF categories, 4.8% of patients had low probability with scores of 0 and 1, and 42.3% were intermediate probabilities (scores 2–4) (Figure 2).

### 3.4. Secondary Outcomes

When NT-proBNP is not applied in the modified HFA-PEFF Diagnostic Algorithm score, results show a different distribution of HFA-PEFF score, with 86.7% of patients having an intermediate probability of HFpEF (Table 4).

Binary logistic regression analysis demonstrated an association between log-transformed NT-proBNP and echocardiographic diastolic dysfunction (OR 1.21, 95% CI 1.00–1.45, *p* = 0.046). However, the overall discriminatory performance of the model was limited, with an AUC of 0.634 and an accuracy of 64.7%. While specificity was high (94.5%), sensitivity remained poor, indicating the limited ability of NT-proBNP alone to discriminate diastolic dysfunction in this CKD cohort.

### 3.5. Exploratory Analysis

Exploratory analysis was performed to evaluate the impact of alternative NT-proBNP thresholds on HFpEF classification and their relationship with echocardiographic diastolic dysfunction. Several cohort-adapted NT-proBNP cutoffs were tested using binary logistic regression and ROC-derived performance metrics. An NT-proBNP threshold of 600 pg/mL showed modest sensitivity (~40%) and specificity (~70%). Among the evaluated exploratory thresholds, a threshold of 700 pg/mL demonstrated a more balanced diagnostic profile, with a sensitivity of 47.5%, specificity of 78.1%, and an AUC of 0.628. In contrast, higher thresholds markedly increased specificity at the expense of sensitivity. Based on these exploratory findings, a cutoff of 700 pg/mL was selected as a cohort-derived threshold for subsequent sensitivity analyses. This threshold should be interpreted as hypothesis-generating rather than as a validated CKD-specific diagnostic cutoff.

When applying the exploratory cohort-derived NT-proBNP threshold of 700 pg/mL in sinus rhythm and 2100 pg/mL in atrial fibrillation, the HFA-PEFF algorithm identified 19.8% patients with scores of 5 and 6, respectively.

### 3.6. Sensitivity Analysis

Sensitivity analyses were performed using alternative echocardiographic definitions of diastolic dysfunction. When diastolic dysfunction was defined using e′ sept < 8 cm/s and/or E′ lateral < 10 cm/s and E/e′ >  9 (prevalence of 52.9%), log-transformed NT-proBNP was no longer significantly associated with diastolic dysfunction (OR 1.09, *p* = 0.336), and discriminatory performance remained poor (AUC 0.55).

## 4. Discussion

In our cohort, more than half of patients with CKD G3–G4 fulfilled criteria for HFpEF using a modified HFA-PEFF diagnostic algorithm and general population biomarker thresholds, despite no prior diagnosis of heart failure. Importantly, this should not be interpreted as overclassification in an otherwise low-risk population. Our patients with CKD G3–G4 displayed a marked cardio-kidney-metabolic phenotype characterized by high rates of metabolic syndrome, arterial stiffness, structural cardiac remodeling, and diastolic dysfunction, supporting the presence of substantial subclinical cardiovascular disease. These findings suggest that cardiovascular involvement in moderate-to-severe CKD is both frequent and clinically significant.

However, the proportion of patients fulfilling modified HFA-PEFF criteria for HFpEF varied substantially according to the NT-proBNP threshold applied, while the discriminatory performance of NT-proBNP for echocardiographic diastolic dysfunction remained modest in this CKD population. Together, these findings suggest that although CKD patients frequently exhibit genuine cardiovascular remodeling and functional abnormalities, biomarker-based HFpEF algorithms may still overestimate or misclassify HFpEF probability in this setting.

In the CPH-CKD ECHO study [14], particularly in CKD stages 3 and 4, diastolic dysfunction was highly prevalent (~48–64%), and LVEF remained preserved (~60%), LVH was relatively modest (~10–14%) and closely linked to albuminuria. In contrast, our cohort showed a higher prevalence of LVH, suggesting a more advanced cardiac phenotype despite lower proteinuria levels. In the CRIC cohort (*n* = 3505), mean LVEF was 54%, with most patients (77%) having preserved systolic function and a low prevalence of overt heart failure (10%). LV mass, geometry, and LVEF were associated with heart failure hospitalization and mortality, although their incremental prognostic value beyond clinical variables was modest [15]. Similarly, our cohort showed frequent structural and diastolic abnormalities, notably interventricular septal hypertrophy (67.8%) and diastolic dysfunction (31.6%). In addition, the high prevalence of left atrial enlargement observed in our cohort may reflect a broader atrial remodeling phenotype frequently encountered in CKD and HFpEF. In patients with preserved left ventricular systolic function, atrial remodeling, atrial fibrillation, and CKD-associated valvular or annular calcification may contribute to atrial functional mitral regurgitation, highlighting the importance of integrating valvular and atrial echocardiographic parameters into the characterization of the cardio-renal HFpEF phenotype [16]. A cross-sectional study in CKD patients (predominantly stages 3–4) reported a mean LVEF of ~50%, with systolic dysfunction present in 24% and diastolic dysfunction in 58% of patients, both increasing with CKD severity [17]. A recent prospective study in CKD (G1–G5) showed that HFpEF incidence increases with declining eGFR and higher albuminuria, reaching 6.9% in advanced stages. Biomarkers of cardiac stretch, fibrosis, and remodeling were strongly associated with HFpEF risk and improved prediction beyond clinical variables, supporting the link between renal dysfunction, myocardial changes, and HFpEF [18].

NT-proBNP is a well-established prognostic biomarker in CKD and has consistently been associated with increased risks of all-cause mortality, major adverse cardiovascular events, and progression to end-stage kidney disease. Prior studies in non-dialysis CKD populations demonstrated that higher NT-proBNP levels are independently associated with adverse cardiovascular outcomes, supporting its role as a marker of cardiovascular risk and subclinical cardiac dysfunction in CKD. Nevertheless, despite its prognostic value, the interpretation of NT-proBNP within HFpEF diagnostic algorithms remains challenging in CKD populations, where structural cardiac remodeling and impaired renal function may substantially influence circulating peptide levels [6]. NT-proBNP-based diagnostic algorithms may substantially influence HFpEF classification in CKD populations, while the discriminatory performance of natriuretic peptides in this setting remains uncertain. Recent studies suggest that kidney function-adjusted NT-proBNP thresholds improve HF risk stratification in CKD populations. Consistent with these findings, we observed marked differences in HFpEF classification according to the NT-proBNP threshold applied [19].

This study has several strengths. First, it provides a detailed structural and metabolic characterization of patients with CKD stages 3–4 without previously diagnosed CV events, highlighting a marked cardio-kidney-metabolic phenotype characterized by high rates of metabolic syndrome, arterial stiffness, cardiac remodeling, and diastolic dysfunction. Second, the study specifically addresses the diagnostic challenges of HFpEF in CKD by evaluating the impact of NT-proBNP thresholds within the HFA-PEFF algorithm. Third, by exploring hypothesis-generating cohort-derived NT-proBNP thresholds and alternative echocardiographic definitions of diastolic dysfunction, our findings emphasize the substantial influence of natriuretic peptide thresholds on HFpEF classification in CKD populations.

This study also has several limitations. Its cross-sectional design precludes causal inference, and the relatively small sample size limits generalizability. Echocardiographic assessment was partly limited by suboptimal acoustic windows and missing data for some parameters, while PWV measurements were unavailable in a subset of patients due to technical and logistical limitations. Additionally, the exploratory NT-proBNP thresholds were derived and evaluated within the same cohort and therefore require external validation before any clinical application. Also, diastolic dysfunction represents only one component of the HFpEF syndrome and may itself be variably expressed in CKD populations.

## 5. Conclusions

Patients with CKD stages G3–G4 exhibited a marked cardio-kidney-metabolic phenotype characterized by structural cardiac remodeling, arterial stiffness, and diastolic abnormalities, supporting the presence of substantial subclinical cardiovascular disease. However, HFpEF classification using modified HFA-PEFF-based approaches varied markedly according to the NT-proBNP threshold applied, while NT-proBNP demonstrated only modest discriminatory performance for echocardiographic diastolic dysfunction. These findings suggest that although cardiovascular involvement is highly prevalent in CKD, current biomarker-based HFpEF algorithms may remain susceptible to overclassification or misclassification in this population, supporting the need for kidney function-adapted biomarker strategies and more refined phenotyping approaches.

## Figures and Tables

**Table 1:** Diagnostic algorithm of HFpEF; values are expressed according to ESC diagnostic thresholds [3]. Major and minor criteria contribute 2 and 1 points, respectively, to the overall score.

Functional/Morphological/Biomarker Criteria		Major Criteria	Minor Criteria
Functional echocardiographic criteria	Septal and lateral mitral annular peak early diastolic velocity (e′)	septal e′ < 7 cm/s or lateral e′ < 10 cm/s	-
	Average septal-lateral E/e′ ratio	average E/e′ ≥ 15	average E/e′ = 9–14
	Tricuspid regurgitation (TR) peak velocity or pulmonary arterial systolic pressure (PASP)	TR velocity > 2.8 m/s PASP > 35 mmHg	-
Morphological echocardiographic criteria	Left ventricular global longitudinal systolic strain	-	GLS < 16%
	Left atrial volume index	LAVI > 34 mL/m^2^	LAVI 29–34 mL/m^2^
	Left ventricular mass index and relative wall thickness	LVMI ≥ 149/122 g/m^2^ and RWT > 0.42	LVMI > 115/95 g/m^2^ or RWT > 0.42 or LV wall thickness ≥ 12 mm
Natriuretic peptides profile in:	sinus rhythm (SR)	NT-proBNP > 220 pg/mL or BNP > 80 pg/mL	NT-proBNP 125–220 pg/mL or BNP 35–80 pg/mL
	atrial fibrillation (AF)	NT-proBNP > 660 pg/mL or BNP > 240 pg/mL	NT-proBNP 365–660 pg/mL or BNP 105–240 pg/mL
Score calculation		2 points	1 point
Interpretation	2–4 points: diastolic stress test or invasive hemodynamic measurements ≥5 points: HFpEF		

**Table 2:** Baseline characteristics—Values are presented as median (interquartile range) for non-normally distributed continuous variables, mean ± standard deviation for normally distributed continuous variables, and number (percentage) for categorical variables. Percentages are calculated based on available data.

Category	Variable	Value
Demographic characteristics	Age, years	68 (59–75)
	Male sex	80 (49.7%)
CKD stage	G3a	55 (29.4%)
	G3b	67 (35.8%)
	G4	65 (34.8%)
Etiology of CKD	Hypertensive nephropathy	74 (39.6%)
	Diabetic nephropathy	55 (29.4%)
	Glomerular disease	36 (19.3%)
	Other causes	22 (11.8%)
Comorbidities	Hypertension	172 (92.0%)
	Diabetes mellitus	78 (41.7%)
	Atrial fibrillation	20 (10.7%)
	Hyperuricemia	55 (29.4%)
	Metabolic syndrome	140 (74.9%)
	Dyslipidemia	76/186 (40.9%)
	Hypothyroidism	11 (5.9%)
	Chronic hepatitis	10 (5.3%)
Laboratory parameters	Serum creatinine, mg/dL	1.74 (1.38–2.44)
	eGFR, mL/min/1.73 m^2^	36 (25–47)
	Hemoglobin, g/dL	12.91 ± 2.00 (*n* = 186)
	NT-proBNP, pg/mL	405.5 (122.1–841.4)
Renal parameters	Proteinuria < 0.5	126/184 (68.5%)
	Proteinuria ≥ 0.5	58/184 (31.5%)
Pulse wave velocity		10.45m/s (IQR 8.8–12.7) (*n* = 170)
Medication	*n* (%)	
SGLT2 inhibitors	99 (52.9%)	
GLP-1 receptor agonists	9 (4.8%)	
ACE inhibitors/ARB	112 (59.9%)	
≥3 antihypertensive drugs	38 (20.3%)	
Beta-blockers	103 (55.1%)	
Mineralocorticoid receptor antagonists	33 (17.6%)	
Lipid-lowering therapy	104 (55.6%)	
Antiplatelet therapy	48 (25.7%)	
Anticoagulant therapy	26 (13.9%)	
Allopurinol	49 (26.2%)	

**Table 3:** Baseline echocardiographic measurements.

Parameter	Elevated (*n*, %)	Valid N
LV Structure		
Interventricular septal hypertrophy	124 (67.8%)	183
Posterior wall hypertrophy	95 (53.1%)	179
LV volume index > 86 mL/m^2^	7 (3.86%)	181
LV volume index > 37 mL/m^2^	6 (3.55%)	169
LV Geometry		179
Concentric hypertrophy	41 (22.9%)	
Concentric remodeling	48 (26.8%)	
Eccentric hypertrophy	21 (11.7%)	
Normal geometry	69 (38.5%)	
LV Diastolic Function		
Elevated filling pressures (E/e′ > 15)	20 (11%)	181
Diastolic dysfunction	59 (31.6%)	187
Left Atrium		
Left atrial enlargement (LAVI > 34 mL/m^2^)	97 (61.8%)	157
Elevated LACI (≥6)	26 (17.9%)	145
Right Ventricle and Pulmonary Circulation		
RA dilation	83 (51.8%)	160
RV systolic dysfunction	23 (12.3%)	187
Elevated PAPS (>35 mmHg)	30 (16.1%)	186
Probability of Pulmonary Hypertension		157
Low probability	132 (84.1%)	
Intermediate probability	22 (14%)	
High probability	3 (1.9%)	

**Table 4:** HFA-PEFF Diagnostic Algorithm without NT-proBNP.

HFA -PEFF Score	*n*	%
0	9	4.8
1	16	8.6
2	25	13.4
3	51	27.3
4	86	46.0

## Data Availability

The datasets used and/or analyzed during the current study are available from the corresponding author on reasonable request.

## Supplementary Materials

The following supporting information can be downloaded at: https://www.mdpi.com/article/10.3390/life16060944/s1, Table S1: Echocardiographic parameters; Table S2: Formulas used.

## Abbreviations

The following abbreviations are used in this manuscript:

ACEi	Angiotensin-converting enzyme inhibitors
AF	Atrial fibrillation
ARB	Angiotensin receptor blockers
BNP	B-type natriuretic peptide
CKD	Chronic kidney disease
CV	Cardiovascular
e′	Peak early diastolic mitral annular velocity measured by tissue Doppler imaging
eGFR	Estimated glomerular filtration rate
E/e′	Ratio of early mitral inflow velocity to early diastolic mitral annular velocity;
GLS	Global longitudinal strain
GLP-1	Glucagon-like peptide-1 receptor agonists
HFpEF	Heart failure with preserved ejection fraction
LACI	Left atrial contractility index
LAVI	Left atrial volume index
LV	Left ventricle
LVEF	Left ventricular ejection fraction
LVMI	Left ventricular mass index
MRA	Mineralocorticoid receptor antagonists
NT-proBNP	N-terminal pro–B-type natriuretic peptide
PAPS	Pulmonary artery systolic pressure
RWT	Relative wall thickness
RV	Right ventricle
SGLT2i	Sodium-glucose cotransporter-2 inhibitors
SR	Sinus rhythm
TR	Tricuspid regurgitation
