# Supplementary material for: HFpEF Diagnosis: A Challenge in CKD with Current Algorithms
Source: Life (Basel). 2026 Jun 3;16(6):944. doi: 10.3390/life16060944 (PMC13301278; doi:10.3390/life16060944)
Supplement: Supplementary file 1 [file life-16-00944-s001.zip › life-4336757-supplementary.pdf]

Supplementary material:

Section A

Table S1. Echocardiographic parameters

**Echocardiographic parameters**						
**Left ventricle**	**Left atrium**	**Left ventricular diastolic function**	**Tissue Doppler imaging**	**Valvular Doppler parameters**	**Right ventricle**	**IVC**
IVSd (mm)	LA area (cm^2)	E wave velocity (cm/s)	Lateral e′ wave velocity (cm/s)	MVA (cm^2)	RVEDA (cm^2)	IVC diameter (mm)
LVPWd (mm)	LAV (mL)	A wave velocity (cm/s)	Lateral a′ wave velocity (cm/s)	PHT (m/s)	RVESA (cm^2)	CI (%)
LVIDd (mm)	LAVI (ml/m^2)	E/A	Lateral s′ wave velocity (cm/s)	Vmax (Ao) (m/s)	RVFAC (%)	
LVIDs (mm)	LACI	DT (cm/s)	Lateral E/e′	ΔPmax (Ao) (mmHg)	TAPSE (mm)	
LVEDV (4C) (mL)			Septal e′ wave velocity (cm/s)	Vmax (TR) (m/s)	Tricuspid S′ wave velocity (cm/s)	
LVESV (4C) (mL)			Septal a′ wave velocity (cm/s)	ΔPmax (TR) (mmHg)	RVOT-prox (mm)	
LVEF (4C) (%)			Septal s′ wave velocity (cm/s)	PAT (ms)		
LVEDV (2C) (mL)			Septal E/e′			
LVESV (2C) (mL)			Average E/e′			

LVEF (%) (biplane)						

IVSd = interventricular septal thickness in diastole; LVPWd = Left ventricular posterior wall thickness at end -diastole; LVIDd= Left ventricular internal dimension at end -diastole; LVIDs= Left ventricular internal dimension at end -systole; LVEDV = left ventricular end-diastolic volume; LVESV = left ventricular end-systolic volume; LVEF = left ventricular ejection fraction; LAD = left atrial diameter; LA area = left atrial area; LAV = left atrial volume; LAVI = Left Atrial Volume Index; LACI = left atrial contractility index; E = early diastolic transmitral flow velocity; A = late diastolic transmitral flow velocity; DT = deceleration time of the E wave; e' = early diastolic myocardial relaxation; a' = active atrial contraction in late diastole; s' = longitudinal LV contraction; MVA = mitral valve area; PHT = pressure half-time; Vmax = maximum velocity; Ao = aortic valve; TR = tricuspid regurgitation; ΔPmax = maximum pressure gradient; RVEDA = right ventricular end-diastolic area; RVESA = right ventricular end-systolic area; RV FAC = right ventricular fractional area change; TAPSE = tricuspid annular plane systolic excursion; Tricuspid S' = Tricuspid annular peak systolic velocity; PAT = Pulmonary Acceleration Time; RVOT-prox =Proximal right ventricular outflow tract; IVC = inferior vena cava; IVC CI = inferior vena cava collapsibility index, 2C: 2 chambers, 4C: 4 chambers

Transthoracic echocardiography protocol

End-systole and end-diastole time points were established using concomitant ECG monitoring.

IVSd, LPWd and LVIDd were measured at end-diastole in parasternal long-axis view on 2D echocardiography. LVIDs was measured at end-systole in parasternal long-axis view. Cut-off values were wall thickness above 9 mm in females or 10 mm in men. LV mass was obtained as a derivate value. LV hypertrophy was defined using sex-specific LVMI cut-offs (≥95 g/m² in women and ≥115 g/m² in men). LVMI (left ventricular mass index) was calculated as LVM/BSA using the Devereux-modified ASE formula. Relative wall thickness was calculated, and hypertrophy was considered eccentric if RWT ≤0.42 or concentric if RWT >0.42. Normal LV geometry was defined as normal LVMI and RWT ≤0.42.

End-diastolic and end-systolic volumes of the left ventricle (LV) are estimated using apical 4-chamber and 2-chamber views. These measurements were used to calculate the left ventricular ejection fraction (LVEF) using Simpson's biplane method as EF = [(LVEDV −LVESV) / LVEDV] × 100%. EF was considered preserved if >50%. LV volumes were indexed to body surface area (BSA), which was calculated using the Mosteller formula. LV dilation was defined as indexed LVEDV > 74 mL/m2 (in men) or LVEDV > 61 mL/m2 (in women).

Left atrial dimensions were obtained in apical 4-chamber views. LA volume was indexed to BSA to obtain LAVI. LA dilation was defined as LAVI>34 ml/m2. LACI was calculated as LAVI/ Septal a' wave velocity. Impaired LA mechanical coupling index was defined as LACI ≥6.

E and A wave velocities and Doppler-derived mitral deceleration time were obtained using pulsed-wave Doppler (PW) in apical 4-chamber views.

TDI measurements were performed in apical 4-chamber views and used to reflect systolic and diastolic LV function. Estimated normal LV filling pressures were defined as an average E/e' ratio <8. LV filling pressures were considered elevated when average E/e' ratio was >15. Further, grade 2 diastolic dysfunction was defined as septal e' wave velocity <8 cm/s and/or lateral e' wave velocity <10 cm/s and E/e'> 9.

Proximal RVOT diameter assesed in parasternal long-axis views was chosen as a measurement of RV size. Dilation of the RV was defined as proximal RVOT > 33 mm. RV function was assesed in apical 4-chamber views and not dedicated RV views when possible. RV FAC was calculated as 100 * [(RVEDA – RVESA)/RVEDA)]. TAPSE was measured using M-mode echocardiography. Tricuspid S' wave velocity was obtained using tissue doppler imaging.

RV longitudinal systolic dysfunction was defined as TAPSE (tricuspid annular plane systolic excursion) <17 mm, FAC (fractional area change) <35%, or S' <9.5 cm/s.

Valvular function was assessed using continuous wave (CW) echocardiography in dedicated views. Beyond measurements of diastolic disfunction at the mitral annulus, these are beyond the scope of the analysis at hand.

Inferior vena cava dimensions and collapsibility were assessed in subcostal views. Cut-off values were IVC diametre > 21 mm.

Section B

The indirect assessment of pulmonary artery pressures and pulmonary hypertension probability were performed by standardised calculations derived from tricuspid valvular measurements and are further detailed in Table S2.

Table S2: Formulas used

**Parameter**	**Formula**
BMI (m^2)	BMI (kg/m^2)= $\frac{weight(kg)}{height(m)^2}$
TyG	LN[fasting triglycerides (mg/dL) × fasting plasma glucose (mg/dL) / 2]

BSA	$\sqrt{\frac{height(cm)x\ weight\ (kg)}{3600}}$
LVM (g)	0.8×[1.04×((LVEDD+IVSd+LVPWd)^3−(LVEDD)^3)]+0.6
LVMI (g/m^2)	$\frac{LVM(g)}{BSA\ (m^2)}$
LAVI (mL/m^2)	$\frac{LAV\ (mL)}{BSA\ (m^2)}$
RWT	$\frac{2xLVPWd}{LVEDD}$
RAP	RAP = 3 mmHg: IVC ≤ 21 mm and collapsibility > 50%<br>RAP = 8 mmHg: intermediate values<br>RAP = 15 mmHg: IVC > 21 mm and collapsibility < 50%
PAPS (mmHg)	4×(TR Vmax)2+RAP

Table S2: Formulas used - BMI = body mass index; BSA = body surface area, calculated using the Mosteller formula; TyG (tryglyceride-glucose index), LVM = left ventricular mass, calculated using the Devereux-modified American Society of Echocardiography formula; LVMI = left ventricular mass index; LAVI = left atrial volume index; RWT = relative wall thickness; RAP = right atrial pressure; PASP = pulmonary artery systolic pressure; LVEDD = left ventricular end-diastolic diameter; IVSd = interventricular septal thickness in diastole; LVPWd = left ventricular posterior wall thickness in diastole; LAV = left atrial volume; IVC = inferior vena cava; TR Vmax = maximum tricuspid regurgitation jet velocity.
